# Case Report of Combined Central and Peripheral Demyelination: Treated With Ofatumumab

**DOI:** 10.1002/iid3.70459

**Published:** 2026-05-28

**Authors:** Lili Liu, Zhigang Zhong, Haijun Wu, Jun Hu, Juanjuan Chen

**Affiliations:** ^1^ Department of Neurology Peking University Shenzhen Hospital Shenzhen China

**Keywords:** chronic inflammatory demyelinating polyneuropathy, combined central and peripheral demyelination, disease‐modifying‐therapy, multiple sclerosis, Ofatumumab

## Abstract

**Background and Objectives:**

Combined central and peripheral demyelination (CCPD) is a rare autoimmune‐mediated disorder characterized by demyelination affecting both the central nervous system and peripheral nervous system. The involvement of the CNS typically manifests as longitudinally extensive transverse myelitis and optic neuritis, whereas PNS involvement commonly presents as chronic inflammatory demyelinating polyneuropathy (CIDP). Clinically, the course of CCPD can vary from monophasic to relapsing‐remitting, leading to complex and diverse symptomatology. There are few studies on the treatment of CCPD. Here we describe two patients diagnosed with CCPD, summarize their clinical features and symptoms, and suggest new approaches to treating CCPD.

**Case Presentation:**

We report two young female patients of CCPD with a relapsing–remitting course. Clinically, initial symptoms of limb numbness and weakness responded to treatment but subsequently recurred alongside visual disturbances. Cerebrospinal fluid analysis via lumbar puncture revealed the presence of oligoclonal bands. Nerve conduction studies indicated significantly reduced motor conduction velocities, prolonged distal latencies, conduction block, and extended F‐wave latencies across multiple nerves. Brain MRI scans identified multiple long‐T2 hyperintense lesions affecting the cortex, subcortical white matter, brainstem, and spinal cord. Acute episodes showed improvement following administration of intravenous immunoglobulin and high‐dose methylprednisolone. During remission, treatment with ofatumumab was initiated, and the patients have since remained stable without further relapses.

**Conclusion:**

CCPD is a rare clinical entity that poses a significant challenge for diagnosis and treatment. Our case suggests that Ofatumumab might serve as a potentially effective alternative for CCPD patients.

## 1 Introduction

Combined central and peripheral demyelination (CCPD) is a rare autoimmune‐mediated disorder characterized by demyelination affecting both the central nervous system (CNS) and peripheral nervous system (PNS) [1]. The involvement of the CNS typically manifests as longitudinally extensive transverse myelitis (LETM) and optic neuritis, whereas PNS involvement commonly presents as chronic inflammatory demyelinating polyneuropathy (CIDP) [2]. In some patients, a history of viral infection or vaccination prior to disease onset may be present, and anti‐neurofascin (NF155) antibodies can be detected in some individuals [3, 4]. Clinically, the course of CCPD can vary from monophasic to relapsing‐remitting, leading to complex and diverse symptomatology.

In 1979, Forrester C and Lascelles RG first reported two cases of multiple sclerosis (MS) combined with inflammatory polyneuropathy [5], but it was not until 1986 that Amit et al. [6] proposed the concept of CCPD. Research indicates that certain individuals diagnosed with CIDP may concurrently present with CNS involvement. Conversely, a portion of patients with MS may demonstrate electrophysiological abnormalities within peripheral nerves [7]. Extensive epidemiological studies on CCPD are currently lacking. In addition, the treatment of CCPD has been investigated primarily through case reports and small cohort studies to date. During the acute stage, high‐dose intravenous methylprednisolone (HDMP) and intravenous immunoglobulins (IVIG) remain the first‐line treatment options. However, successful treatment cases with anti‐CD20 antibodies, such as rituximab, have also been published.

In this case report, we present two patients diagnosed with CCPD. The main clinical manifestations were limb weakness and numbness followed by decreased vision. During the remission phase, the patients is undergoing treatment with ofatumumab (OFA), which is another fully human anti‐CD20 monoclonal, and maintaining a stable condition. Our case is notable for the present of characteristic CCPD manifestation, and OFA may be a potentially effective therapy for it.

## 2 Case 1 Presentation

The patient, a 36‐year‐old Han young female, was admitted to our hospital in 2023 due to a history of recurrent vision loss spanning over a decade. In 2006, the patient experienced a sudden decline in visual acuity in her left eye and was subsequently diagnosed with optic neuritis at an external hospital. Concurrently, comorbid hyperthyroidism was also identified. After treatment with oral prednisone and propylthiouracil, there was improvement in symptoms; however, the patient's left eye vision acuity and visual field impairment could not recover completely. After that, the patient did not receive any sequential therapy.

In 2022, the patient experienced acute symmetric sensorimotor deficiency of all four extremities and right facial paralysis. She sought medical attention at another hospital where a lumbar puncture examination was performed. CSF examination revealed white blood cells, 2 × 10^6^/L; protein, 1524 mg/L. ELISAs were performed for the detection of ganglioside antibodies GM1, GQ1B and sulfatide antibodies, all of which were negative. Nerve conduction study (NCS) revealed an obvious reduction in motor conduction velocity, prolongation of F‐wave latency and abnormal temporal dispersion. The compound muscle action potentials (CMAPs) in the right facial nerve also decreased significantly. These findings were consistent with the diagnostic criteria for CIDP (multifocal acquired demyelinating sensory and motor neuropathy subtype, MADSAM). Brain and cervical magnetic resonance imaging (MRI) was normal. Therefore, IVIG 2 g/Kg and HDMP (D1‐4 1000 mg/d, D5‐8 500 mg/d, D9‐12 250 mg/d, D13‐15 120 mg/d), was administered, resulting in neurological improvement.

**Figure 1:**
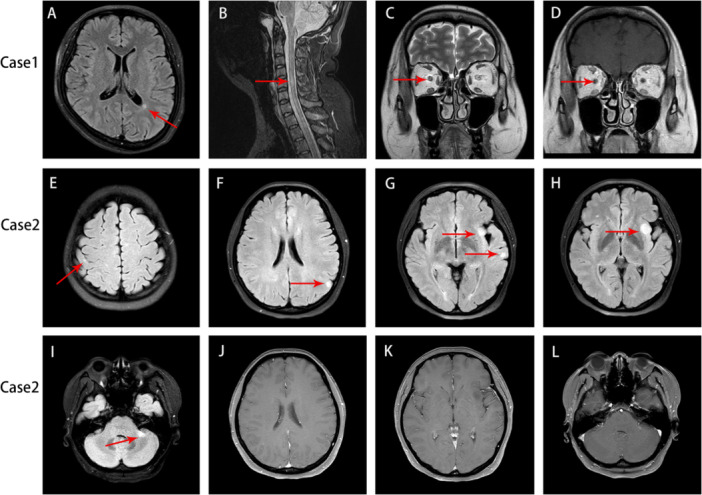
Case 1: Axial fluid‐attenuated inversion recovery (FLAIR) MRI sections revealing hyperintense lesions in the left periventricular regions and cervical segment (A and B). The orbital MRI showed anomalous T2 signals in both optic nerves, with a particular emphasis on the left eye (C and D). Case 2: Cranial MRI revealed abnormally hyperintense signals on FLAIR sequences in the left parietal lobe, left insula, temporal lobe, pontine brachium, left pons, and right frontal lobe (E–I) and no significant enhancement was observed (J–L).

In January 2023, the patient arrived at our hospital with blurred vision in the right eye. Fourteen days earlier, she had acquired a minor instance of coronavirus disease 2019 (COVID‐19), which was verified by a rapid COVID‐19 antigen test. The results of ocular examination were as follows. Vod FC/30 cm (no improvement in correction); Vos 0.2. Slit lamp microscopy revealed a pale boundary of both optic discs. The optic coherence tomography (OCT) scan revealed a decrease in the thickness of the retinal nerve fiber layer (RNFL) in the left eye (Supporting Information S1: Figure S1). The pattern visual evoked potential (VEP) exhibited a prolonged latency of the P100 wave and a decrease in amplitude of both eyes. The Humphrey Visual Field Test (HVF) indicated an enlargement of the physiological blind spot in her right eye, and a significant visual field deficiency in her left eye, particularly in the inferior nasal region. The neurological examination revealed hyporeflexia in both lower limbs and sensory disturbance distributed in a stocking pattern (Expanded Disability Status Scale EDSS = 3.5). The laboratory assessment examination: the routine and biochemical tests conducted on the CSF analysis yielded normal results. The presence of oligoclonal bands (OCB) in CSF was found, with more than two bands being observed. The results of the cell‐based assay (CBA) for anti‐AQP4 antibodies, anti‐MOG antibodies, anti‐GFAP antibodies, and anti‐nodal/paranodal proteins antibodies in both serum and CSF were negative. The MRI of the brain and spinal cord revealed the presence of T2 and flair periventricular and cervical spinal hyperintense lesions, which did not show any enhancement. The orbital MRI showed anomalous T2 and enhanced signals in both optic nerves, with a particular emphasis on the right eye (Figure 1). The NCS still revealed a reduction in the velocity of sensorimotor conduction and motor conduction block, which affected several nerves. Treated with another cycle of high dose IVIg and HDMP, significant improvement was observed in the patient's visual acuity (Vod 0.8 and Vos 0.25). Neurological examination residued bilateral hyporeflexia in the lower extremities and paresthesia in the left toe (EDSS = 1.5).

At the 1‐month follow‐up, the patient's clinical state exhibited continuous improvement. Specifically, the visual acuity of the right eye was 1.0, visual field defect was relieved, and there was no paresthesia. Then prednisolone was tapered and it was decided to begin disease‐modifying‐therapy (DMT). The administration of OFA was recommended owing to the repeated involvement of the optic nerve, which had a significant negative influence on the patient's quality of life. Additionally, there was an increased proportion of peripheral CD19+ cells (25.04%, with a normal range of 5.00%–18.00%). Over a duration of 1 year, the patient's clinical symptoms remained stable. The visual acuity was maintained at 1.0 in the right eye and 0.6 in the left eye. The HVF test revealed an expansion of the typical blind spot in the left eye, with no abnormalities observed in the right eye. The neurological examination revealed hyperreflexia and mild hypoesthesia in the left leg (EDSS = 1.5). The percentage of peripheral CD19 + B cells remained consistently low, at 0.02%. Additionally, the NCS still revealed a reduction in the velocity of sensorimotor conduction (Table 1).

**Table 1:** Case 1: A summary of NCS. The NCS of bilateral median, ulnar, peroneal, and tibial nerves revealed a severe reduction of motor conduction velocity and prolongation of F‐wave latency. ND: Not done.

Motor NCS														
			Year of examination											
Nerve	Site		Latency (ms)				Amplitude (mV)				Conduction velocity (m/s)			
			2022	23‐1‐13	23‐5‐17	23‐9‐27	2022	23‐1‐13	23‐5‐17	23‐9‐27	2022	23‐1‐13	23‐5‐17	23‐9‐27
Median	Right	Wrist	2.6	2.4	ND	2.5	14.4	13.6	ND	13.9	/	/	/	/
		Elbow	8.2	8.80	ND	8.0	12.4	10.3	ND	11.1	33	48.0	ND	45
	Left	Wrist	2.6	ND	2.6	2.6	11.8	ND	9.7	14.2	/	/	/	/
		Elbow	7.9	ND	8.0	8.8	9.6	ND	8.5	12.1	34	**ND**	42.9	50.0
UInar	Right	Wrist	2.3	2.0	ND	2.2	9.3	11.2	ND	12.0	/	/	/	/
		Elbow	10.1	6.10	ND	6.1	9.5	8.70	ND	10.7	34	39.0	ND	38.0
		Above elbow	18.80	9.40	ND	8.9	2.3	8.20	ND	9.6	**ND**	45	ND	49.0
	Left	Wrist	2.1	ND	2.4	2.1	8.6	ND	8.6	12.1	/	/	/	/
		Elbow	9.5	ND	5.0	8.5	7.8	ND	7.4	10.5	31	ND	35.7	38.0
		Above elbow	16.9	ND	10.1	2.1	2.4	ND	7.6	11.9	ND	ND	46.4	51.3
Peroneal	Right	Ankle	3.3	3.1	ND	2.9	3.2	ND	ND	7.1	/	/	/	/
		Fibular head	14.1	12.30	ND	11.9	0.4	3.60	ND	4.0	35.0	33.0	ND	34.0
		Popliteal	ND	14	ND	13.3	ND	4.10	ND	4.4	ND	53.0	ND	59.0
	Left	Ankle	3.1	ND	3.2	3.0	4.4	ND	5.5	8.2	/	/	/	/
		Fibular head	12.2	ND	10.9	12.1	4.0	ND	4.3	6.8	31.0	ND	40.3	40
		Popliteal	ND	ND	13.7	13.8	ND	ND	2.4	6.6	ND	ND	26.8	55
Tibial	Right	Ankle	4.3	3.4	ND	3.6	4.1	5.0	ND	11.7	/	/	/	/
		Popliteal	19.3	14.20	ND	13.5	1.4	10.0	ND	6.1	26.0	**33.0**	**ND**	33
	Left	Ankle	4.6	ND	4.7	4.0	4.3	ND	7	14.9	/	/	/	/
		Popliteal	20.4	ND	14.0	14.3	2.4	ND	3.6	7.0	23.0	**ND**	**33.0**	31

**Sensory NCS**														
						**Year of examination**								
						**Peak latency (ms)**			**Amplitude (μV)**			**Conduction velocity (m/s)**		
**Nerve**			**Site**			**2022**	**23‐1‐13**	**23‐5‐17**	**2022**	**23‐1‐13**	**######**	**2022**	**23‐1‐13**	**23‐5‐17**
Median			Right			2.1	2.7	2.1	22.3	10.7	15.8	59.0	65.0	61.0
Ulnar			Left			2.3	1.7	1.8	11.9	10.0	7.0	50.0	60.0	62.9

**Late responses**														
	**Latency (ms)**											
	**2022**						**2023‐9‐1**					
R tibial F‐wave			50.1						62.2					
L tibial F‐wave			77.2						56.5					

## 3 Case 2 Presentation

The patient, a 44‐year‐old Han female, was admitted to our hospital in 2024 due to numbness in the limbs for over a year, with recurrence and worsening for more than 7 days. The patient developed numbness at the tip of the left little finger without an obvious cause over 1 year ago (May 2023). The symptoms gradually progressed to weakness and numbness in the left hand. Prior to October 2023, the patient experienced numbness and weakness in the right lower limb, accompanied by a sensation of “walking on cotton wool,” foot drop, and frequent tripping. Subsequently, on October 20, 2023, the patient sought medical evaluation in our department. Neurological examination after admission revealed muscle strength Grade 4 in the right lower limb, diminished reflexes in both upper limbs, and absent reflexes in both lower limbs. The laboratory assessment examination: the routine and biochemical tests conducted on the CSF analysis yielded normal results. OCBs were detected in both serum and CSF, with a higher number of OCBs in the CSF compared to the serum. Nerve conduction studies revealed obvious reduction in motor conduction velocity, most notably across Erb's point in the upper limbs. Proximal CMAP amplitudes were reduced by 39%–50% or more compared to distal amplitudes, indicating motor conduction block. No definitive abnormalities were detected in the F‐wave study of the left tibial nerve. Cranial MRI conducted with and without the administration of contrast agents demonstrated normal findings. Based on the patient's symptoms, signs, and examination results, the diagnosis is considered to be multifocal acquired demyelinating sensory and motor neuropathy (MADSAM). Therefore, IVIG 0.4 g/Kg and HDMP (D1‐3 1000 mg/d, D4‐6 500 mg/d, D7‐9 250 mg/d, D10‐12 120 mg/d), was administrated. The patient's limb numbness and weakness had completely resolved, and prednisone acetate was maintained at a dose of 10 mg every other day (Table 2).

**Table 2:** Case2: A summary of NCS. Nerve conduction studies revealed obvious reduction in motor conduction velocity, most notably across Erb's point in the upper limbs. Proximal CMAP amplitudes were reduced by 39%–50% or more compared to distal amplitudes, indicating motor conduction block. ND: Not done.

Motor NCS											
			Year of examination								
Nerve	Site		Latency (ms)			Amplitude (mV)			Conduction velocity (m/s)		
			2023	24‐11‐19		2023	24‐11‐19		2023		24‐11‐19
Median	Right	Wrist	ND	2.7		ND	9.5		/		/
		Elbow	ND	6.90		ND	8.5		/		57.0
	Left	Wrist	2.7	2.5		11.3	9.6		/		/
		Elbow	6.6	6.4		8.9	7.0		62		59.0
UInar	Right	Wrist	ND	1.7		ND	6.2		/		/
		Below Elbow	ND	4.40		ND	6.10		/		61.0
		Above elbow	ND	6.80		ND	5.50		**/**		56.0
	Left	Wrist	2.5	2.0		8.5	5.3		/		/
		Below Elbow	5.3	4.7		5.2	4.4		54		57.0
		Above elbow	7.7	6.8		3.3	4.3		52		55.0
Peroneal	Right	Ankle	3.8	3.8		4.1	3.4				/
		Fibular head	11.8	11.20		1.0	3.40				39.0
		Popliteal	15.2	13		1.0	3.00				31.0
	Left	Ankle	3.3	ND		4.5	ND				/
		Fibular head	10.2	ND		4.2	ND				46.0
		Popliteal	11.6	ND		4.0	ND				57.0
Tibial	Right	Ankle	3.9	3.8		15.3	15.0				/
		Popliteal	11.7	11.90		7.7	5.6				**44.0**
	Left	Ankle	3.5	ND		15.6	ND				/
		Popliteal	11.1	ND		11.1	ND				49.0

**Sensory NCS**											
					**Year of examination**						
					**Peak latency (ms)**			**Amplitude (μV)**		**Conduction velocity (m/s)**	
**Nerve**			**Site**		**2023**		**24‐11‐19**	**2023**	**24‐11‐19**	**2023**	**24‐11‐19**
Median			Left		2.9		2.7	36.9	14.1	56.0	60.0
Ulnar			Left		2.6		2.2	11.8	12.4	52.0	66.0

**Late responses**											
			**Latency (ms)**								
			**2023**					**2024‐11‐19**			
R median F‐wave			/					26.0			
L median F‐wave			41.3					26.8			
R tibial F‐wave			58.1					52			
L tibial F‐wave			44.2					/			

In November 2024, the patient was re‐admitted to our hospital due to sudden onset of decreased vision and visual field defects. And gradually developed numbness in the left hand, but after the first episode resolved completely, there has been no limb numbness since. The uncorrected visual acuity in both eyes is 0.08. The visual fields in both eyes were significantly narrowed, but the fundus and intraocular pressure (IOP) were normal. Neurological examination revealed brisk reflexes in both upper limbs and diminished reflexes in both lower limbs. The VEP exhibited a prolonged latency of the P100 wave and a decrease in amplitude of both eyes. Repeat lumbar puncture re‐examination showed normal results in routine and biochemical analysis. OCBs were detected in both serum and CSF, with a higher number of OCBs in the CSF compared to the serum. The results of CBA for anti‐AQP4 antibodies, anti‐GFAP antibodies, and peripheral neuropathy antibodies in both serum and CSF were negative. But the anti‐MOG antibody in the CSF is positive (1:10). Nerve conduction studies revealed obvious reduction in motor conduction velocity. Proximal CMAP amplitudes were reduced compared to distal amplitudes, indicating motor conduction block. No definitive abnormalities were detected in the F‐wave study of the left tibial nerve. The latency of the F‐wave in the right tibial nerve was significantly prolonged. Biopsy of the right sural nerve revealed mild peripheral neuropathy with a mild reduction in myelinated nerve fibers and mild demyelination. Cranial MRI revealed abnormally hyperintense signals on T2 and FLAIR sequences in the left parietal lobe, left insula, temporal lobe, pontine brachium, left pons, and right frontal lobe. Based on the above findings, this patient was ultimately diagnosed with combined central and peripheral demyelinating disease. Therefore, methylprednisolone pulse therapy was administered during hospitalization. At discharge, the limb numbness symptoms had improved compared to before, but significant visual acuity decline remained. The visual acuity of the right eye was 0.5 and the left eye was 0.3. The DMT involves OFA therapy. Over a duration of 6 months, the patient's clinical symptoms remained stable.

## 4 Discussion

CCPD is a rare demyelinating disease characterized by significant clinical heterogeneity. The available literatures primarily comprises case reports and a limited number of small cohort studies. A Japanese study, which included pediatric patients, reported an age of onset ranging from 8 to 59 years, with an average of 31 years [8]. In contrast, Chinese and Italian cohort studies revealed higher average ages of onset, specifically 47 and 57 years, respectively [9]. All this suggests that CCPD can occur at any age, though it primarily affects young and middle‐age adults. The majority of the patients exhibit a relapsing‐remitting or chronic progressive clinical courses, with only a minority experiencing a monophasic course. Individuals with sequential involvement of the CNS or PNS are more likely to have a relapsing‐remitting course compared to those with simultaneous involvement of both systems [10]. Notably, Case 1 had a prior infection with the novel coronavirus (SARS‐CoV‐2) before experiencing the third episode. Approximately 10%–65% of CCPD patients had a antecedent history of vaccination or infection, with viral infection being the predominant culprit responsible for demyelination. Over the past several years, SARS‐CoV‐2 has become a worldwide pandemic and cases of CCPD following SARS‐CoV‐2 infection or vaccination have been documented [2]. The SARS‐CoV‐2 spike protein attaches to sialic acid‐containing glycoprotein and gangliosides on the surface of cells, leading to the activation of antibodies and resulting in damage to both central and peripheral nerves [10].

The clinical presentations of CCPD are diverse. According to research, 87% of patients in Europe predominantly present with bilateral lower limb weakness and abnormal urinary and bowel function, which are indicative of severe spinal cord injury [7]. In the Japanese cohort, paresthesia (94.9%), limb weakness (92.5%), and mobility disturbance (79.5%) were the most commonly symptoms, which is consistent with the results of China, respiratory disorders, coma, abnormalities in mental behavior, and epilepsy are infrequent [8, 9]. There are two distinct subtypes of CCPD: involvement predominately of the PNS and involvement predominately of the CNS. Certain individuals with predominant PNS involvement exhibit a preponderance of positive NF155 antibodies, which frequently manifest as tremor, distal acquired demyelinating symmetric neuropathy (DADS), and ataxia. Furthermore, visual impairments were observed in 15.4%–47.5% of these cases, with a greater percentage displaying subclinical impairment in VEP. Hence, the utilization of VEP examinations to identify individuals with CCPD can be beneficial for CIDP, especially those are positive in neurofascin (NF)−155 (NF‐155) antibody tests [3]. The patients who exhibit predominant involvement of the CNS may satisfy the diagnostic criteria for MS, neuromyelitis optica spectrum disorders (NMOSD), or MOGAD. In the event that the patient presents with distal limb paresthesia, root pain, and absent tendon reflexes, it is prudent to contemplate the potentiality of a PNS injury [7, 8, 9]. Further analysis of NCS might help identify subclinical peripheral nerve demyelinating lesions. In our study, all patients exhibited symptoms of limb weakness, numbness, and impaired vision. Additionally, cerebrospinal fluid analysis in one case revealed the presence of MOG antibodies.

NCS suggest that the most common finding in patients with CCPD is a decrease in motor nerve conduction velocity, with the peroneal, tibial, and median nerves being the most affected. The proximal segments are more severely involved than the distal segments. Between 27% and 42% of patients may exhibit conduction block and temporal dispersion. The disappearance of F‐wave and H‐reflex, prolongation of latency, and other abnormalities are more common in Asian patients [8]. Slowing of sensory nerve conduction velocity and reduction in amplitude may also occur. Abnormalities in VEP potentials are helpful in detecting subclinical lesions or for follow‐up and efficacy assessment. The electrophysiological examination results of the patient in our cases were consistent with the above characteristics.

MRI of CCPD reveals demyelinating lesions in the cerebral hemispheres, cerebellum, brainstem, and spinal cord. However, the proportion of abnormal optic nerve MRI findings is relatively small, possibly due to insufficient clinical application of this examination. Lesions in the cerebral hemispheres are primarily distributed in the cortex, subcortex, and periventricular area, similar to MS [7, 8, 9]. Spinal cord lesions are primarily located in the cervical and thoracic segment [7, 8]. Ogata H reported on 40 CCPD patients, dividing them into two groups: those with simultaneous CNS and PNS involvement (symptoms appearing within 2 months of each other) and those with temporarily separated onset of CNS and PNS involvement [8]. Comparison of MRI findings between this two groups revealed that patients with simultaneous CNS and PNS involvement were more likely to exhibit diffuse demyelinating lesions in the brain (lesion diameter > 3 cm) and longitudinally extensive spinal cord lesions (LESCLs, > 3 vertebral segments), with proportions of 62.5% and 37.5%, respectively. Conversely, patients with temporarily separated onset of CNS and PNS involvement tended to have peripheral nerve damage superimposed on isolated spinal cord lesions. This may suggest different pathogenetic mechanisms between the two subgroups. Given the high incidence of LESCLs in CCPD patients (37.5%–60%), it is essential to consider this as a significant differential diagnosis for diseases primarily manifesting as LESCLs [7]. Additionally, the proportion of enhancing lesions in CCPD is relatively low, but individual patients may exhibit enhancement in specific areas such as the meninges, spinal nerve roots, and conus. In our study, Case 1 was primarily distinguished by imaging findings indicative of optic nerve involvement accompanied by concurrent spinal cord lesions. In contrast, Case 2 demonstrated multiple lesions within the cerebral cortex, subcortex, and brainstem. Consequently, it is advisable for patients with CCPD presenting with optic neuritis to undergo early orbital MRI and cranial MR examinations, as these modalities can enhance the evaluation of lesion presence and distribution.

The clinical manifestations of CCPD are highly heterogeneous. Current evidence mainly comes from case reports and small sample studies. If a patient presents with CNS manifestations (such as blurred vision, diplopia) and PNS symptoms (e.g., limb weakness, facial paralysis) in the same or sequential disease episodes, CCPD should be highly suspected. Research indicates that CCPD patients often have multiple intracranial lesions and long‐segment transverse myelitis. Thus, those with extensive spinal or cerebral lesions should be evaluated for CCPD [8, 9]. MRI findings in CCPD patients frequently show notable hypertrophy, thickening, and elevated T2 signal intensity in the brachial or lumbosacral plexus, indicating peripheral nerve involvement. Thus, CCPD should be considered in patients with CNS demyelinating lesions and nerve root thickening and hypertrophy [11]. The underlying pathophysiological mechanism of CCPD remains elusive. Some studies propose that CCPD is a distinct spectrum disease, which cannot be merely regarded as an additive combination of MS and CIDP [9]. The pattern of CNS involvement in CCPD patients is atypical for MS [12]. Many studies have suggested that a majority of CCPD cases are preceded by infection, indicating that exogenous infections may induce humoral immune responses and thereby promote autoimmune neural injury [9, 13]. Research has demonstrated that in patients with CCPD, lumbar puncture analysis of cerebrospinal fluid reveals protein‐cell dissociation, elevated protein levels, and an increased cell count exceeding 5 cells/μL, indicative of an inflammatory response. Nonetheless, OCB tests yielded negative results, aligning with prior studies and suggesting that the pathogenesis of CCPD is distinct from that of MS [8, 9, 14]. Consequently, several studies propose that certain exogenous antigens, including proteins derived from viruses or vaccines, may exhibit structural similarities with common targets within the CNS and PNS, such as myelin‐associated proteins. This structural mimicry may precipitate a cross‐reactive immune response, culminating in concurrent damage to both central and peripheral nerves. Moreover, specific neuronal membrane proteins, such as antibodies against NF‐155, NF‐186, and MOG proteins, are expressed in both the CNS and PNS. The production of autoantibodies against these proteins by the immune system may result in the involvement of both systems [2, 12, 15, 16, 17]. For patients with central or PNS demyelinating diseases who test positive for NF155, NF186, and MOG antibodies, the possibility of CCPD should be considered.

Since CCPD is an immune‐mediated demyelination disease, available immunotherapies such as corticosteroid, plasma exchange (PE), and IVIG are employed. In cases where initial therapies prove ineffective, anti‐CD20 monoclonal antibody, rituximab may be considered [7, 8, 18, 19, 20]. Recently, one case has been published with ocrelizumab having been successfully used in a patient with CCDP who suffered from both MS and CIDP [18]. OFA is a second generation anti‐CD20 monoclonal antibody that targets the CD20 antigen at a novel binding site. It has reduced allergenicity, slower dissociation rates, and enhanced complement‐dependent cytotoxicity compared to rituximab [21]. The efficacy of OFA has been particularly been reported in patients with relapsing‐remitting MS [22]. Our patients presented with recurrent visual impairment, necessitating long‐term disease‐modifying treatment to prevent recurrences. Following discussion with the patients, they opted for OFA therapy due to its greater convenience. After a 6‐month follow‐up period, the patients’ condition remained stable, exhibiting improved visual acuity without any clinical recurrences. The treatment and follow‐up of the two patients are ongoing.

This study has certain limitations. Firstly, pathological examination of peripheral nerves was not performed in the first case. In addition, our study was based on only two cases, representing an inherently small sample size. This limitation restricts the generalizability of our findings and precludes definitive conclusions about the efficacy and safety of OFA in the broader CCPD population. Lastly, the follow up spanned only several months to several years and is still ongoing, so the long‐term prognosis and treatment efficacy remain unclear.

## 5 Conclusion

In summary, CCPD is a rare autoimmune neurological disorder with varied clinical features and unclear pathogenesis and immune traits. Our cases contribute valuable insights into the management of this complex disorder, however, it is crucial to acknowledge the inherent limitations posed by the small sample size. Furthermore, there is a pressing need for large‐scale randomized controlled trials and extended follow‐up periods to thoroughly assess long‐term outcomes. The rarity of CCPD, coupled with its intricate pathophysiological mechanisms, necessitates further research to delineate optimal therapeutic strategies and enhance the understanding of this enigmatic condition.

## Author Contributions

Writing – original draft: Lili Liu and Juanjuan Chen. Writing – review and editing: Juanjuan Chen and Jun Hu. Supervision: Juanjuan Chen and Jun Hu. Resources: Zhigang Zhong and Haijun Wu. Data Curation: Zhigang Zhong and Haijun Wu. Conceptualization: Lili Liu and Juanjuan Chen.

## Disclosure

AI was not used in this article.

## Ethics Statement

The study was reviewed and approved by the Medical Ethics Committee of Peking University Shenzhen Hospital (case2026‐004).

## Consent

Written informed consent was obtained from the patients for publication of the case report and any accompanying images.

## Conflicts of Interest

The authors declare no conflicts of interest.

## Supporting information

Supporting Figure

## Data Availability

The original data presented in the study are included in the article. Further inquiries can be directed to the corresponding author.
