# Supplementary material for: Case Report of Combined Central and Peripheral Demyelination: Treated With Ofatumumab
Source: Immun Inflamm Dis. 2026 May 28;14(5):e70459. doi: 10.1002/iid3.70459 (PMC13240514; doi:10.1002/iid3.70459)
Supplement: Supplementary file 1 — Supporting Figure [file IID3-14-e70459-s001.docx]

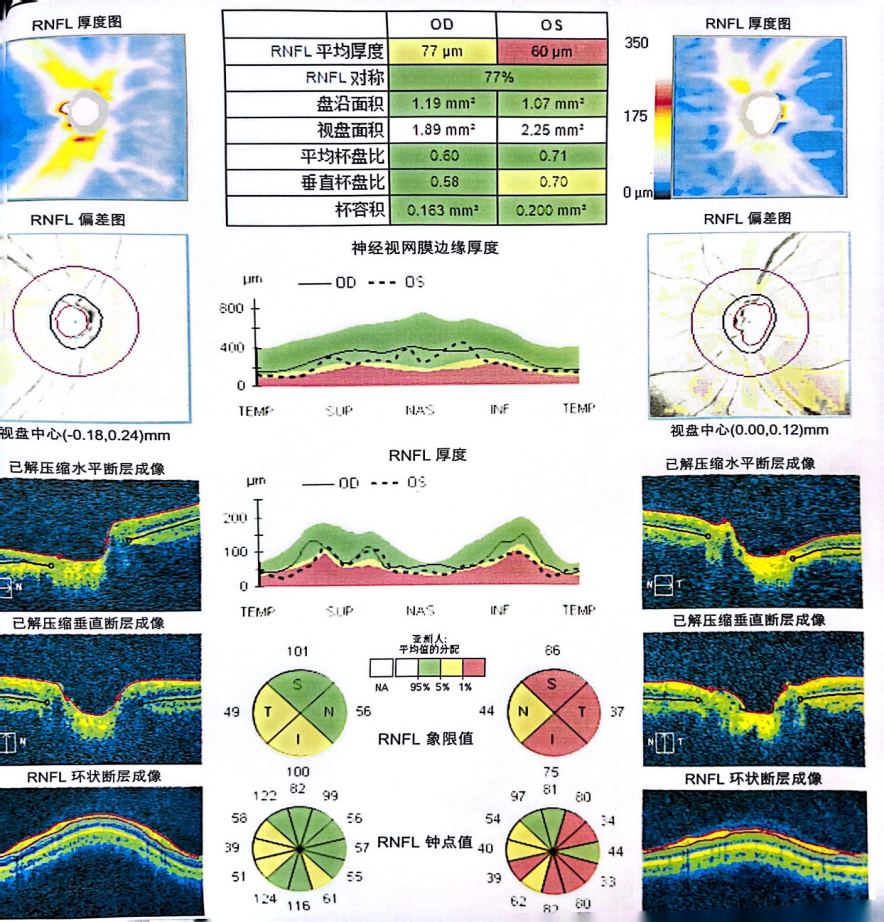


**Supplement Fig. 1**. Abnormal OCT demonstrated moderate RNFL thinning in the right inferior and temporal quadrant, while severe thinning over the left superior, inferior and temporal quadrant (average thinkness right 77μm, left 60μm)
